# Beyond ADCs: harnessing bispecific antibodies to directly induce apoptosis for targeted tumor eradication

**DOI:** 10.1093/abt/tbae029

**Published:** 2024-10-29

**Authors:** Victor S Goldmacher, Iosif M Gershteyn, Yelena Kovtun

**Affiliations:** Research and Development Department, ImmuVia, Inc., Cambridge, MA 02142, United States; Research and Development Department, ImmuVia, Inc., Cambridge, MA 02142, United States; Research and Development Department, ImmuVia, Inc., Cambridge, MA 02142, United States

**Keywords:** apoptosis, bispecific antibody, death receptor 5, targeted cancer therapy, TNFRSF10B

## Abstract

Bispecific apoptosis triggers (BATs) are innovative bispecific antibodies designed to simultaneously target both a tumor-associated antigen and a cancer cell’s death receptor, thereby directly activating the extrinsic apoptotic pathway to induce death of cancer cells. This unique mechanism distinguishes BATs from antibody-drug conjugates (ADCs), which rely on cytotoxic drugs, and bispecific immune cell engagers such as bispecific T-cell engagers (BiTEs) and bispecific natural killer cell engagers (NKCEs), which recruit immune cells to eliminate target cancer cells. BATs offer significant potential advantages in clinical efficacy and safety over ADCs and BiTEs. Although the field is still emerging, recent advancements are highly promising, and analysis of preclinical and clinical data of DR5-targeting antibodies have been pivotal in outlining the criteria for the next generation of effective and safe medicines. Antibodies found inactive in preclinical testing were also found to be clinically ineffective, whereas antibodies with minimal preclinical results demonstrated moderate clinical activity. All clinical DR5-targeting antibodies were well tolerated by patients even at high doses (with the exception of TAS266 due to its unique design). These findings underscore the predictive value of robust preclinical models on clinical outcomes. Notably, first-in-class BAT, Cancerlysin™ IMV-M, demonstrated potent efficacy in diverse xenograft cancer models and safety in non-human primates, marking a significant advancement in developing safe and effective anti-cancer drugs.

## Teaser

Statement of Significance: Bispecific apoptosis triggers (BATs) target tumor cells, inducing apoptosis without harming healthy tissues. BATs potentially overcome limitations of antibody-drug conjugates, may benefit wider patient populations than ADCs, and are less toxic. With these advantages, BATs pave the way for effective and safe cancer therapies.

## Introduction

With the advent of monoclonal antibodies [1], extensive research focused on developing cancer therapies targeting antigens expressed on the surface of cancer cells. The initial concept was that upon binding to a cancer cell, an antibody would either clear the cell from circulation, in the case of blood malignancies, or recruit immune cells through its Fc portion thereby leading to the destruction of the tumor cell via antibody-dependent cellular cytotoxicity (ADCC), antibody-dependent cellular phagocytosis (ADCP), or complement-dependent cytotoxicity (CDC). However, these initial hopes went largely unrealized, especially in the context of solid tumors. In many cases, monoclonal antibodies did little more than bind to the cancer cells and hence failed during clinical development. The exceptions were antibodies that could directly damage cancer cells by interacting with functional receptors such as EGFR or HER2. This realization led to a shift in strategy: for most targeted antibodies to be effective in eradicating tumors they needed to be armed or otherwise modified to carry a cytotoxic function [2].

The subsequent generation of therapeutic antibodies which recognized tumor-associated antigens (TAAs) but did not function effectively as naked antibodies focused on enhancing anti-tumor activity by equipping them with additional mechanisms of action to improve their ability to target and destroy cancer cells. Two major strategies for arming antibodies were explored: one approach involved attaching cytotoxic proteins to the antibody, creating immunotoxins. These proteins were typically highly potent enzymes that interfered with protein synthesis, aiming to kill cancer cells upon immunotoxin internalization [3]. However, with several exceptions, immunotoxins failed in clinical trials. The primary issue was their high toxicity, which often led to severe side effects. In addition, immunotoxins provoked immune responses (immunogenicity) that rendered repeated doses ineffective due to the rapid development of neutralizing antibodies. Finally, immunotoxins short circulation half-life limited sustained efficacy against tumors and resulted in the need for frequent intravenous administration in clinics.

Another major approach of arming antibodies is by attaching highly cytotoxic small molecules thus creating antibody-drug conjugates (ADCs) [4]. ADCs work by binding to specific antigens on the cell surface and then being internalized through endocytosis. Once inside the cell, the ADC is trafficked to the lysosome where the antibody component is degraded, releasing the cytotoxic payload intended to kill the cancer cell from within. Initially, these conjugates utilized conventional chemotherapeutic drugs as their payloads. However, it became apparent that these drugs were not potent enough to be effective at the levels delivered inside the cell. The breakthrough came alongside the understanding that the payload needed to be much more potent than traditional chemotherapy. This realization led to the development of a new generation of ADCs, starting with the first publication on a prototype of Kadcyla® (Trastuzumab emtansine) [5]. These advanced ADCs quickly gained traction in the field, leading to the approval of many ADCs that since have become successful treatments for a variety of cancers [6] Despite their successes, ADCs have significant limitations: First, they are only effective for a subset of patients—those with high expression of the target antigen; without sufficient antigen expression insufficient drug is delivered to achieve therapeutic efficacy, as seen, for example, with Elahere™ (mirvetuximab soravtansine) [7]. Second, the maximum tolerated dose of ADCs in patients is constrained by the inherent toxicity of the payload, which can lead to severe side effects. Third, many patients who have undergone extensive pretreatment with other therapies develop resistance [8, 9], which may extend to the payload classes used in ADCs. This resistance is often reflected in the low overall response rates (ORR) observed in Phase 1 trials involving heavily pretreated patients. Finally, the manufacturing process for ADCs is both complex and costly, requiring the separate production of the antibody and the cytotoxic drug, followed by conjugation, which contributes to the expense of the final product.

Other promising approaches include bispecific immune cell engagers, such as BiTEs and NKCEs, which facilitate the engagement of immune cells to kill target cells [10], and the related approach of chimeric antigen receptor T-cell (CAR-T) therapy [11]. A detailed discussion of these approaches is beyond the scope of this review, but it is important to note that, to date, these therapies have primarily been effective in treating blood malignancies. However, these approaches present significant challenges when applied to solid tumors due to the complex immunosuppressive environment within tumors, and are associated with severe side effects, such as cytokine release syndrome.

Therefore, there is a critical need for new approaches in targeted therapy that are more effective, less toxic, and more cost-efficient.

In this review, we introduce a novel approach that we have named bispecific apoptosis triggers (BATs). BATs are bispecific antibodies that target a tumor-associated antigen (TAA) on cancer cells and a death receptor, aiming to directly activate the extrinsic apoptotic pathway in cancer cells in a TAA-selective manner leading to cell death. The difference in the mechanism of eradicating target cells distinguishes BATs from ADCs, which rely on toxic drug payloads, and from bispecific antibodies that engage immune cells to kill target cells. BATs aim to address the limitations of previous cancer therapies by combining several advantageous features: (i) they selectively target TAAs, (ii) require no toxic drug payloads or internalization for efficacy, thereby overcoming the two major limitations of ADCs, (iii) induce cell death through a mechanism distinct from those of ADCs and chemotherapeutic drugs, circumventing resistance mechanisms that may have developed from prior chemotherapy or ADC therapy, (iv) do not recruit immune cells, thus retaining activity in the immunosuppressive tumor microenvironment and avoiding the risk of cytokine release syndrome (which poses challenges for bispecific T-cell engagers and CAR-T therapies), (v) finally, the manufacturing process for BATs is straightforward and well-established, akin to that of conventional IgG antibodies.

## Recognizing death receptors as targets for cancer therapy

With the discovery that tumor necrosis factor (TNF) can kill cells [12], and the identification of its receptor, TNF Receptor 1, the search for therapeutics that induce apoptosis (programmed cell death) in cancer cells began. However, in clinical trials, TNF caused significant inflammatory toxicity [13] and its clinical development was abandoned. Later, several other TNF receptor superfamily members were identified, including Fas/CD95, death receptor 4 (TNFRSF10A; DR4), and death receptor 5 (TNFRSF10B, TRAILR2, or DR5) [14].

Apoptosis induced by death receptors is mediated through the extrinsic apoptotic pathway. When a death receptor on the cell surface binds to its ligand, such as Fas ligand or TRAIL, it triggers receptor clustering, which subsequently facilitates the recruitment and activation of pro-caspase-8 or pro-caspase-10. Once activated, these initiator caspases cleave and activate downstream effector caspases, leading to the degradation of cellular substrates and ultimately resulting in apoptotic cell death characterized by DNA fragmentation, membrane blebbing, and cell disassembly [14].

Targeting Fas was discontinued after it was found that an anti-Fas antibody induced sudden and severe hepatotoxicity in mice [15]. In contrast, early research indicated that TRAIL, the ligand for both DR4 and DR5, and its recombinant derivative, recombinant soluble APO2L/TRAIL, were safe and did not cause systemic toxicity, while effective at inducing apoptosis in cell lines derived from a wide variety of cancers and promoting the regression of xenograft tumors in immunodeficient mice [16, 17]. This discovery spurred the development of therapeutic agents aimed at targeting DR4 and DR5 for cancer treatment.

## Early struggles: monospecific predecessors targeting death receptors

**Figure 1:**
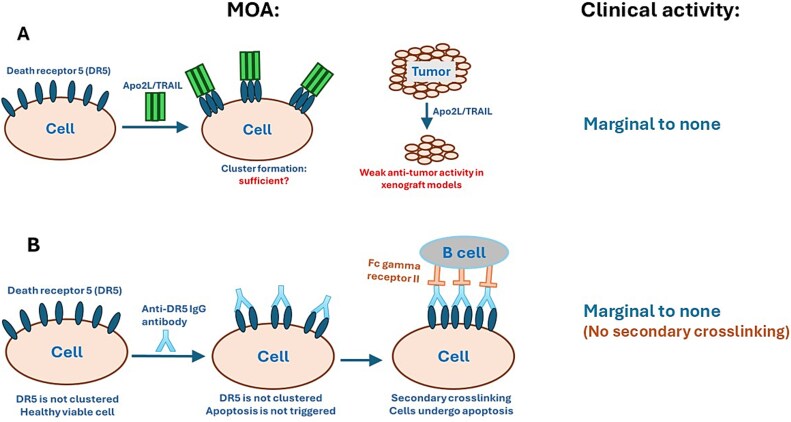
Schematic representation of initial clinical attempts to use recombinant TRAIL, its derivatives, and IgG antibodies targeting DR4 or DR5 as anti-cancer drugs. (a) Targeting cancer cells with APO2/TRAIL. Its mode of action (MOA), the crosslinking of DR5, may be limited to clustering only three DR5 molecules. The lack of effective crosslinking of DR5 is likely a major reason for the poor or absent anti-tumor activity of these agents in patients. (b) IgG anti-DR5 antibodies, being bivalent, are unable to cluster DR5 without secondary crosslinking. In nude mice, mouse B cells provide this secondary crosslinking by engaging IgG via their Fc gamma receptor II. In human patients, this secondary crosslinking may not be efficient enough to eradicate tumors because (i) unlike young nude mice used in experiments, humans have a very high concentration of their own IgG in blood, blocking the Fc gamma receptor II, and (ii) tumor infiltration by B cells in patients may be insufficient.

A recombinant soluble trimer derivative of TRAIL, Apo2L/TRAIL (developed by Genentech) was able to kill various cancer-derived cell lines *in vitro*, which was considered promising [18]. However, preclinical activity in xenograft models demonstrated only weak responses: even after up to ten injections of high doses (10 to 120 mg/kg) of Apo2L/TRAIL, only modest effects were observed in established xenograft tumors [16, 17, 19–21]. This poor preclinical anti-tumor activity of Apo2L/TRAIL could, at least in part, be attributed to its limited clustering ability, possibly restricting it to forming only trimers of DR5 (Fig. 1a), and/or to its short circulation life (minutes), which limits exposure to the tumor. Not surprisingly, in clinical trials, Apo2L/TRAIL, while safe for patients, demonstrated only marginal antitumor activity. Why was there such a discrepancy between the strong *in vitro* cytotoxicity of Apo2L/TRAIL and its limited *in vivo* activity? In retrospect, the *in vitro* cytotoxic activity of Apo2L/TRAIL might have been largely driven by a small fraction of its aggregates inducing super-clustering of the death receptors, whereas *in vivo*, such aggregates were likely cleared rapidly from circulation. This notion is supported by observations that an artificially cross-linked version of Apo2L/TRAIL has a much higher ability to trigger apoptosis than the original Apo2L/TRAIL [22].

Another TRAIL derivative, Circularly Permuted Tumor Necrosis Factor-Related Apoptosis-Inducing Ligand (CPT), also known as aponermin, demonstrated moderate anti-tumor activity in a xenograft model even after multiple large doses, and required being given in combination with a chemotherapeutic agent to achieve a more pronounced effect [23]. In a Phase 1 and Phase 2 clinical studies CPT was well-tolerated in patients and demonstrated moderate clinical activity as a single agent despite a short circulation half-life of ~1 h [24–27]. A Phase 3 trial of aponermin in combination with thalidomide and dexamethasone demonstrated the combination to be moderately more active than thalidomide/dexamethasone alone [28]. Aponermin was approved for use in combination with thalidomide and dexamethasone for the treatment of patients with relapsed or refractory multiple myeloma in November 2023 in China [29]. This newer TRAIL derivative is still under investigation and represents a significant step forward in the therapeutic landscape for relapsed/refractory multiple myeloma but has had limited success overall.

Following mixed results with targeting death receptors using Apo2L/TRAIL, researchers focused on agonistic antibodies that target either DR4 or DR5 to induce apoptosis. Seven IgG anti-death receptor monoclonal antibodies have been tested in clinical trials: one anti-DR4 and six anti-DR5. The preference for DR5 targeting is due to accumulating evidence that DR5 expression in cancers is more universal and at higher levels than that of DR4 and that DR5 is overexpressed in the majority of cancerous tissues compared to normal tissues [30–41]. Data from the Broad Institute’s human gene expression database (https://depmap.org/portal) indicate that DR5 expression is high and comparable across a vast majority of cell lines derived from diverse cancers. As reported in [30] (Supplement), nearly all examined cancer-derived cell lines exhibited significant DR5 levels, with many displaying plasma membrane DR5 expression exceeding 1x10^4^ molecules per cell when data were available. Notably, even cell lines expressing as few as 7 × 10^3^ or 1.2 × 10^4^ DR5 molecules per cell were sensitive to a TRAIL derivative targeting death receptors, despite having lower DR4 levels. These findings suggest that even relatively low DR5 levels can be sufficient to activate the extrinsic apoptotic pathway. While some cell lines showed resistance, the underlying factors contributing to this resistance were not explored, and this resistance did not correlate with DR5 expression levels. One major mechanism involves decoy death receptors [17], which are also recognized by TRAIL. Similarly, as highlighted in [42], most tested clinical tumor samples and cancer-derived cell lines exhibited high DR5 expression and were sensitive to apoptosis induced by a bispecific anti-DR5 antibody, further supporting DR5 as a viable therapeutic target in oncology. Collectively, these findings suggest that DR5 is expressed at levels sufficient to induce apoptosis upon activation in most cancerous tissues in a wide range of cancers.

Mapatumumab, also known as HGS-ETR1 or TRM1 [31], is the only anti-DR4 monoclonal antibody that has been evaluated in clinical trials. However, none of the clinical trials of mapatumumab met their initial objectives, leading to the discontinuation of its clinical development (reviewed in [43]). Six antibodies targeting DR5—lexatumumab, conatumumab (AMG655), drozitumab (Apomab), DS-8273a, tigatuzumab, and LBY135—have been evaluated in clinical settings. While all were found to be safe at very high doses (dose-limiting toxicity at 20 mg/kg or higher, if reached at all), their efficacy results have been disappointing [44–51]. The decisions to take anti-DR4 and anti-DR5 antibodies into clinical trials were based on preclinical studies showing promising results. These preclinical activities were puzzling since IgG antibodies are bivalent and do not bring together more than two molecules of a death receptor (Fig. 1b), and thus would be expected to be unable to crosslink DR5 in the absence of secondary crosslinking. Crosslinking of these antibodies with secondary antibodies (reviewed in [43]) markedly enhanced their ability to induce DR5-mediated apoptosis. It was convincingly shown in mouse models that the anti-tumor activity of IgG anti-death receptor antibodies was driven by secondary crosslinking via their interaction with the Fc gamma receptor II (FcγRII), found mainly on B cells [52, 53] (Fig. 1b). When the antibodies were mutated to inhibit their interaction with FcγRII, they lost their anti-tumor activity *in vivo* completely. In human patients, this secondary crosslinking was apparently insufficient, which may explain why these antibodies lacked anti-tumor activity in clinical trials. While B cells apparently infiltrated tumors in the reported experiments with nude mice, human B cells infiltrate patients’ tumors only sparingly or inconsistently [54–57]. In addition, while young nude mice, typically used in preclinical studies, lack antibodies in their blood (e.g., [58]), humans have high levels of IgG antibodies in their blood [59], saturating FcγRII and inhibiting the secondary crosslinking of the therapeutic antibodies.

## Enhanced crosslinking for improved therapeutic efficacy: second-generation DR5-targeted therapies

The initial clinical attempts to use recombinant TRAIL, its derivatives, and agonistic antibodies targeting DR4 and DR5 have been largely disappointing. As noted, to trigger apoptosis death receptors must be clustered [60, 61], but the methods to achieve this for practical applications in drugs, and the degree of clustering required, were not well established. Therefore, an empirical approach to find effective agents was taken.

Ablynx and Novartis developed TAS266, a molecule combining four identical nanobodies against DR5 into a single entity via a linker [62]. TAS266 was reported to have diverse anti-tumor activity in a few primary patient-derived colon and pancreatic tumor xenografts, although the extent of this activity is unclear due to the brief and indirect description of the experimental results. TAS266 turned out to be intolerably hepatotoxic to patients and its development was terminated. However hepatotoxicity was only observed in patients with preexisting antibodies to TAS266 [63], suggesting that the tetravalent molecule was not inherently toxic without additional secondary crosslinking of more than four DR5 molecules by the patient’s antibodies.

Inhibrx developed INBRX-109, a tetravalent antibody composed of two identical anti-DR5 nanobodies in tandem, fused to the N-terminus of human IgG1 Fc portion, which was silenced to prevent interaction with Fc-gamma receptors. In preclinical studies, the anti-tumor activity of INBRX-109 was reported in numerous *in vitro* models, but only in three xenograft models. It showed moderate activity in two patient-derived xenograft models and good anti-tumor activity in COLO205 xenografts in mice [64] [US Patent 10,308,720], a model known to be highly sensitive in the hands of many researchers. Thus, the preclinical results did not demonstrate robust and diverse anti-tumor activity. One plausible explanation for the limited preclinical activity of INBRX-109 is that clustering of only four DR5 molecules may not be optimal for inducing apoptosis. However, in a Phase I study, INBRX-109 was well tolerated and demonstrated moderate antitumor activity, which was compelling enough to initiate a Phase II study [65].

Eftozanermin α (ABBV-621) was designed as an IgG1 Fc fusion linked to two sets of the natural trimeric TRAIL, thus potentially crosslinking six DR5 molecules. This agent was highly active across multiple tumor cell lines in sub-nanomolar range *in vitro*, demonstrated antitumor activity in numerous PDX models with multiple injections, and was well tolerated in cynomolgus monkey [30]. In line with the preclinical observations, ABBV-621 was safe and showed signs of activity in a Phase 1 clinical trial [66].

IGM Biosciences developed IGM-8444, an IgM antibody with 10 potential binding sites to DR5 [67]. Despite multiple doses, IGM-8444 showed only partial effects in three xenograft models, including the highly sensitive COLO205, necessitating the use of drug combinations to enhance activity. A potential reason for this limited efficacy is the rigidity of the pentameric IgM geometry, which may not allow optimal clustering of DR5. Consequently, the preclinical results did not demonstrate robust and diverse anti-tumor activity. In the ongoing clinical trial, interim data indicates that the antibody is safe [68]. Possibly due to concerns about its efficacy as a single agent, the phase 1 trial was conducted in combination with other drugs, and these combinations have shown some clinical activity, including partial responses. Drug combination rather than single-agent use makes it difficult to estimate the degree of contribution of IGM-8444 to the anti-tumor activity in patients.

Genmab developed HexaBody-DR5/DR5 (GEN1029), an equimolar mixture of two IgG1 antibodies that bind to non-overlapping epitopes of DR5. These antibodies feature an Fc-domain mutation that enhances antibody hexamerization after binding to the cell surface target. Together, hexamerization and binding to non-overlapping epitopes were intended to create conditions for extensive DR5 clustering on the surface of tumor cells [69]. GEN1029 demonstrated preclinical *in vivo* activity in numerous cancer models. However, the Phase 1 clinical trial NCT03576131 was terminated at a low dose due to high toxicity, suggesting that GEN1029 indiscriminately attacked not only tumors but also normal tissues in patients.

Thus, experience with agents targeting DR5 supported the value of preclinical *in vivo* data: those agents without sufficient preclinical *in vivo* activity turned out to lack activity in the clinic, while those which only demonstrated modest preclinical activity showed moderate clinical activity in clinical trials, some—promising enough, at least in drug combinations. By extension, this correlation between the extent of preclinical and clinical activity predicts that agents with robust preclinical activity would be active in clinical trials as well. Additionally, results with monospecific agents targeting DR5 revealed a catch-22: if DR5 is crosslinked efficiently, it would result in the death of normal cells (making the treatment unsafe). Conversely, if DR5 is crosslinked moderately to avoid harming normal cells, the cancer cells would not be effectively eliminated (making the treatment safe but not very potent). With that in mind, researchers turned to searching for agents that would recognize cancer cells and then trigger apoptosis only in these cells.

## Challenges of initial bispecific apoptosis triggers: insufficient death receptor clustering

The initial attempts to selectively induce apoptosis in cancer cells focused on fusing TRAIL derivatives to various molecules, primarily antibodies or their fragments targeting tumor-associated cell surface molecules (Table 1). These bispecific TRAIL derivatives were tested in a limited number of *in vitro* models and even fewer *in vivo* models. Almost none progressed to clinical trials. This approach was largely abandoned.

**Table 1:** TRAIL-containing BATs

Targeting molecule	Targeted cells	References
Fn14 (targets TWEAK)	Hepatocellular carcinoma	[76]
EGFR-targeting (several fusion proteins)	EGFR-positive solid tumors	[77–82]
Anti-CD19	CD19-positive B cell malignancies	[83]
Anti-CD33scFv-TRAIL	Myeloid leukemia	[84]
Melanoma-associated chondroitin sulfate proteoglycan (MCSP)-TRAIL	Melanoma	[85]
Anti-CD40-TRAIL	CD40-expressing B-cell malignancies and solid tumors	[86]
Anti-CD70-TRAIL	CD70-positive blood malignancies	[87]
ACDCRGDCFC peptide (named RGD-L-TRAIL), a ligand of alpha(V)beta(3) and alpha(V)beta(5) integrins.	Tumor microvasculature	[88]
Anti-Kv10.1 (a voltage-gated potassium channel)-TRAIL	Kv10.1-positive tumors	[89]
ANTI-multidrug resistance protein 3 (MRP3)-TRAIL	MPR3-positive tumors	[90]

**Figure 2:**
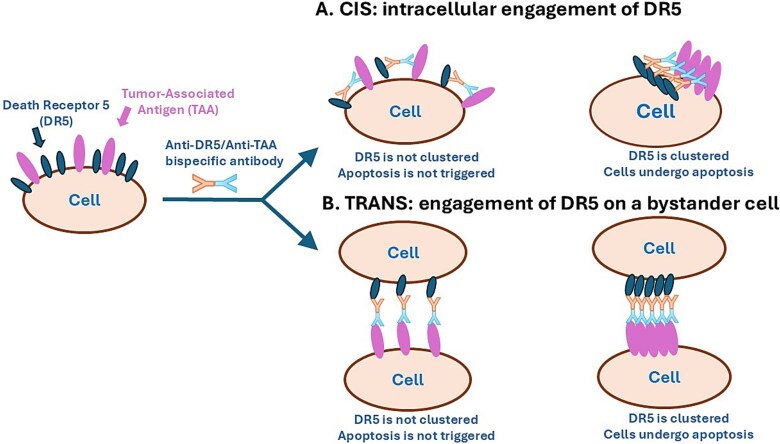
Schematic representation of the interaction between BATs and tumor cells. (a) BAT engaging TAA and DR5 on the same cell (*cis* engagement). *In vitro* cytotoxicity experiments with sparsely plated cells may be limited to only this mode of apoptosis induction. (b) BAT engaging DR5 on a cell adjacent to the TAA-expressing target cell (*trans* engagement, or bystander killing). *In vivo* experiments with tumor cells packed together in xenografts (along with stromal cells) may involve both *cis*- and *trans*-modes of action. Apoptosis is triggered only if the BAT can provide DR5 clustering, not just mere binding. Results with several BATs have demonstrated that tumor-selective engagement of DR5 can be achieved. However, first-generation BATs may not have provided sufficient clustering of DR5 for massive apoptosis of cells within a tumor, as reflected by modest clinical results. The next generation of BATs aims to overcome this problem.

The next approach focused on developing BATs that utilize an anti-DR5 antibody or its fragment as the warhead (Table 2). The hope was that TAA would provide for crowding of BAT molecules on the cell surface, which would then cause the DR5-binding regions of the BAT to promote DR5 clustering and cancer cell death (Fig. 2a). Shivange et al. [70] described a BAT targeting folate receptor 1 (FOLR1), a TAA that is overexpressed in several cancers. The bispecific antibody, designated BaCa, exhibited highly selective cytotoxicity toward cultured cell lines in a FOLR1-specific manner, with cytotoxicity more than two orders of magnitude greater than that of the anti-DR5 monospecific antibody (one of its components). BaCa was also potent in eradicating FOLR1-expressing xenograft tumors in nude mice. Interestingly, BaCa induced apoptosis both in the FOLR1-expressing cell and in an adjacent cell, resulting in the killing of FOLR1-negative cells in mixed cultures of FOLR1-positive and FOLR1-negative cells (bystander cytotoxicity). This suggests that BaCa could eradicate tumors with heterogeneous FOLR1 expression (via *trans*-induction of apoptosis, as illustrated in Fig. 2b). Importantly, the *in vivo* anti-tumor activity of this antibody required secondary crosslinking via FcγRII: a BaCa mutant with impaired FcγRII interaction completely lost its *in vivo* anti-tumor activity. In this respect, BaCa was similar to monospecific anti-DR5 antibodies. The lack of *in vivo* anti-tumor activity of BaCa in the absence of secondary crosslinking suggests that the degree of FOLR1-mediated crowding of BaCa is insufficient to cluster DR5 and to induce apoptosis without an additional stimulus for crosslinking. To date, BaCa does not appear to have reached the stage of human clinical testing.

**Table 2:** BATs containing an anti-DR5 antibody derivative

Targeting molecule	Targeted cells	References
Anti-FOLR1	Ovarian cancer, endometrial cancer, non-small cell lung cancer, and breast cancer	[70]
Hyaluronate polysaccharide targeting receptor for hyaluronan-mediated motility (RHAMM) and CD44	Variety of solid tumors and normal liver	[91]
Anti-MCSP	Melanoma	[92]
Anti-lymphotoxin-beta receptor (LTβR)	Epithelial tumors	[93]
Anti-cadherin17 (CDH17)	Cancers of GI tract	[42, 71]
Anti-FAP	Stromal cells in solid tumors; trans-anti-tumor cell activity	[73, 74]

Two BATs were developed with the implicit hope that TAAs with multiple epitopes on their surface, recognized by a targeting BAT, would facilitate effective crosslinking of the BAT. This crosslinking was intended to generate sufficient clustering of DR5, thereby promoting the apoptosis of the target cells. García-Martínez et al. [42] reported BI 905711, a bispecific antibody targeting cadherin-17 (CDH17), which is overexpressed in several cancers, and DR5. CDH17 has seven homologous segments (repeats) in its extracellular domain. There is no published evidence indicating whether BI 905711 binds to one or more of these repeats of BI 905711, and whether more than one BI 905711 molecule can bind to the same CDH17 molecule. However, an illustration on the Boehringer Ingelheim website suggests this possibility, although no descriptive text accompanies the image. BI 905711 was tested in five *in vivo* xenograft models. In all cases, there was poor separation in tumor growth time course between the control (non-treated mice) and the mice treated with BI 905711, making the assessment of its anti-tumor potency challenging. To enhance the response, a combination treatment of BI 905711 with irinotecan was required. BI 905711 was completely safe in monkey toxicity studies. Not surprisingly, in phase 1 clinical trial, BI 905711 was found to be safe and modestly active [71], and later the agent was tested in combination with chemotherapy in clinic [72]. This unconvincing preclinical activity and only modest clinical activity may stem from insufficient ability of BI 905711 to cluster DR5 on cancer cells: amino acid sequence alignment of the repeats reveals low homology among them, and only between two repeats at a time. Consequently, it is likely that the antibody binds to no more than two repeats simultaneously, but even this has not been explicitly reported. Importantly, BI 905711 activated DR5 on both CDH17-positive tumor cells and adjacent tumor cells, demonstrating bystander cytotoxicity activity [42].

The bispecific antibody RG7386 (RO6874813) is a variation on this theme. It targets fibroblast-activation protein (FAP) on cancer-associated fibroblasts in tumor stroma and DR5 on tumor cells, again demonstrating the capacity of BATs to induce bystander cytotoxicity (Fig. 2b) [73]. FAP is a homo-dimeric membrane protein, and the hope was that upon encountering RG7386, multiple FAP protein molecules would be crosslinked, supporting the clustering of DR5 on the other end of RG7386. Its anti-cancer activity in preclinical *in vivo* studies was unimpressive: RG7386 required multiple high-dose injections to slow down tumor growth, therefore a combination with irinotecan or doxorubicin was required to improve activity. In a phase 1 trial RO6874813 was safe, but its anti-tumor activity in patients was underwhelming [74]. This can be attributed to two possible explanations: (i) insufficient clustering of DR5 and/or (ii) not every tumor cell being in the vicinity of a stromal cell. Once again, lackluster preclinical activity was a good predictor of weak clinical activity.

Several essential questions regarding the efficacy of BATs as anti-cancer agents still need to be explored further as the field evolves.

The first is determining the required level of TAA expression for effective BAT activity. Given that DR5 clustering in BATs is TAA-dependent, this may necessitate TAA expression above a certain threshold, as suggested by findings in [70]. However, drawing definitive conclusions is premature due to the limited data currently available.

As with any form of chemotherapy, the question of emerging resistance, is also crucial. A key concern is whether resistance to apoptosis will be acquired following DR5-targeting therapy. This is an important issue, but since the BAT approach is still in its early stages, no answer is available yet. However, one aspect can already be addressed: cell death suppressor genes, when overexpressed, can increase cell resistance to extrinsic apoptosis (which is triggered by DR5). These include members of the BCL-2 family, such as BCL2, BCL-XL (BCL2L1), and MCL1; the IAP family, including XIAP, c-IAP1, c-IAP2, BIRC6, and survivin; as well as FLIP, heat shock proteins like HSP70 and HSP27, TNFAIP3, and AKT1. An analysis of the expression of these genes in ~1500 cancer-derived cell lines from the Broad Institute’s human gene expression database (https://depmap.org/portal/) shows that, in most of these cell lines, expression levels of these genes are comparable to each other. This suggests that their expression is not significantly elevated, implying that resistance to apoptosis has not yet emerged in the tumors from which these cell lines originated. Future clinical studies with next-generation BATs will provide more definitive insights into whether the resistance to apoptosis develops after BAT therapy and how frequently these resistant tumors occur.

The format of the DR5-targeting molecule may significantly influence its activity and safety profile. Currently, insufficient data were generated, and too few BAT formats have been investigated to draw definitive conclusions. Thus far, the BATs tested in clinical trials have demonstrated a favorable safety profile and modest activity.

Finally, the relative affinities of a BAT for the TAA and DR5 may impact its selectivity. At present, data are available for only three BATs (Table 3), making it premature to draw any conclusions.

**Table 3:** Affinities of BATs to the TAA and DR5

BAT	TAA K_D_	DR5 K_D_	Assay	Reference
Anti-FOLR1/anti-DR5	12 nM	3 nM	Bio-layer interferometry	[70]
Anti-CDH17/anti-DR5	30 nM	200 nM	Surface plasmon resonance	[42]
Anti-FAP/anti-DR5	1.5 nM	0.3 nM	Cell-based	[73]

## Conclusion and recent advances

The development of BATs and their monospecific predecessors has faced significant challenges. Although results with several BATs have demonstrated that tumor-selective engagement of DR5 can be achieved, early agents were either inactive or only moderately effective in preclinical cancer models and, unsurprisingly, underperformed in clinical trials—leading to skepticism about the approach. The early underperformance could be largely attributed to insufficient clustering of death receptors on cell surfaces by these agents, resulting in inefficient triggering of apoptotic cell death. A significant breakthrough came recently with the development of Cancerlysin™, ImmuVia’s proprietary BATs designed to efficiently cluster death receptors through a unique mechanism, greatly enhancing their ability to promote apoptosis in cancer cells. The flagship Cancerlysin™ IMV-M has demonstrated impressive and diverse anti-tumor activity in xenograft tumor models in mice and safety in non-human primates. The manuscript describing this BAT and its mechanism of action is in preparation.

BATs offer several attractive features compared to conventional ADCs:

1. Distinct mechanism of action: BATs operate through mechanisms unrelated to chemotherapeutic drugs and ADCs, thus avoiding chemotherapy-acquired resistance.
2. No requirement for internalization: unlike ADCs, which require endocytosis by tumor cells to be effective, BATs act on the DR5 receptor present on the cell surface. This circumvents the limitation of ADCs; only benefitting patients with high TAA expression necessary for sufficient payload delivery to kill tumor cells. BATs are designed to help a broader patient population.
3. Simplified manufacturing: the production of BATs involves a standard recombinant manufacturing process, with expression levels comparable to those of conventional IgG1 monoclonal antibodies, unlike the complex and expensive manufacturing required for ADCs.
4. Enhanced tolerability: BATs do not employ toxic compounds, avoiding the payload-related toxicities associated with ADCs. Based on accumulated experience, BATs are expected to be well tolerated by patients, even at very high doses.
5. Bystander cytotoxicity activity: similar to ADCs with a cleavable linker [75], and unlike mono-specific anti-DR5 agents, BATs exhibit bystander cytotoxicity activity. They can kill tumor cells via *trans*-clustering of DR5 on neighboring cells, potentially enhancing their anti-tumor activity and enabling the eradication of tumors with heterogeneous TAA expression [42, 70, 73].

With these advantages, we believe BATs are transforming the field of targeting death receptors in tumors, paving the way for highly effective and safe cancer therapies.

## Conflict of interest statement

All authors are employees of ImmuVia, Inc.

## Ethics and consent statement

Consent was not required.

## Animal research statement

Not applicable.

## Data Availability

No new data were generated or analyzed in support of this research. Data supporting the information provided here are all from the public literature or public websites.
